# Nanoscale Control of Carrier Transport in Monolayer Transition-Metal Dichalcogenide Double Heterostructures

**DOI:** 10.1021/acs.nanolett.6c00504

**Published:** 2026-05-08

**Authors:** Jinpeng Tian, Guangming Cheng, Jingtao Tan, Satya Butler, Yin Liang, Haining Mao, Jaehoon Ji, Jaerin Kim, Nan Yao, Saien Xie

**Affiliations:** † Department of Electrical and Computer Engineering, 6740 Princeton University, Princeton, New Jersey 08544, United States; ‡ Princeton Materials Institute, 6740 Princeton University, Princeton, New Jersey 08544, United States; § Department of Mechanical and Aerospace Engineering, Princeton University, Princeton, New Jersey 08544, United States

**Keywords:** 2D materials, transition metal dichalcogenides, lateral double heterostructures, thermionic transport, electron tunneling

## Abstract

Heterostructures are fundamental to modern electronics and optoelectronics. Lateral heterostructures of two-dimensional (2D) semiconductors provide a promising platform for monolayer device architecture. However, the carrier transport mechanisms across these lateral heterointerfaces, especially in heterostructures with nanometer-scale dimensions, remain underexplored. Here, we report the synthesis of monolayer transition-metal dichalcogenide lateral double heterostructures (LDHs) with coherent, dislocation-free interfaces and sub-10 nm dimensional control, including WS_2_–MoS_2_–WS_2_ and WS_2_–WSe_2_–WS_2_. Using WS_2_–WSe_2_–WS_2_ LDHs as a model system, we investigate the electron transport mechanism across the WSe_2_ barrier and observe a transition from thermionic emission to direct tunneling as the WSe_2_ width decreases to sub-10 nm. Importantly, the effective barrier height can be modulated by the gate voltage and source-drain bias, enabling electrostatic control of charge injections. These findings establish LDHs as a powerful platform for engineering transport within monolayer semiconductors, offering new opportunities for next-generation 2D electronic and quantum devices.

## Graphical abstract

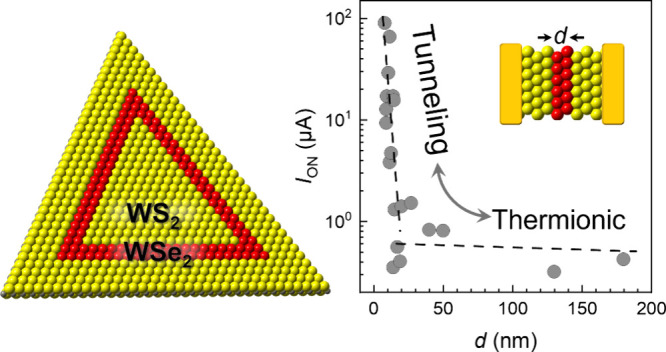

Heterostructures enable precise control over carrier transport and recombination through composition engineering.^1^ Connecting two heterojunctions in series leads to double heterostructures—a critical device structure that has enabled key technologies such as bipolar junction transistors and efficient light-emitting diodes.^2,3^ Realizing such heterostructures in two-dimensional (2D) materials, where distinct monolayer semiconductors are joined seamlessly within a monolayer plane, presents a compelling platform for next-generation electronics.^4,5^ Transition metal dichalcogenides (TMDs) have emerged as promising candidates due to their excellent electronic performance and efficient electrostatic gate control.^6,7^ Their unique band structures further enable direct band gaps,^8,9^ large excitonic effects,^10,11^ and valleytronics,^12,13^ allowing for emerging device applications. However, most monolayer TMD heterostructure devices to date have been based on single heterojunctions without nanometer-scale in-plane compositional modulation, which limits the development of complex device architectures and functionalities.^14^ To unlock the full potential of 2D materials, it is essential to achieve lateral integration of distinct monolayer semiconductors with nanometer-precise control over compositions and dimensions, and to develop a fundamental understanding of the carrier transport in such nanometer-precise lateral heterostructures.

Existing approaches, however, have only produced lateral 2D heterostructures with limited dimensional control, and the carrier transport mechanisms in these heterostructures remain underexplored. For example, patterned regrowth techniques relying on lithographic processes and separate growth steps often lead to an incoherent interface due to contamination and thermal cycling.^15–22^ One-pot synthesis approaches typically suffer from limited control over precursor delivery and temperature stability for different TMD growths, resulting in limited compositional or dimensional control.^14,23–38^

Here, we report the synthesis of TMD lateral double heterostructures (LDHs) with compositional and dimensional control with sub-10 nm precision, including WS_2_–MoS_2_–WS_2_ and WS_2_–WSe_2_–WS_2_. Atomic-resolution imaging confirms that the interfaces in these heterostructures are coherent without misfit dislocations. Using WS_2_–WSe_2_–WS_2_ LDHs as a model system, we demonstrate that electron transport across the WSe_2_ barrier (width noted by *d*) transitions from thermionic emission at large WSe_2_ widths to direct tunneling as the WSe_2_ width decreases to sub-10 nm. Furthermore, we show that the effective barrier height for electron injection can be tuned by applying gate voltage and source-drain bias.

In a TMD LDH, two distinct TMDs are laterally connected in the monolayer plane to form a TMD1-TMD2-TMD1 structure (Figure 1a). Scanning electron microscopy (SEM) images of representative TMD LDHs (Figure 1b-d) show distinct contrast among MoS_2_, WS_2_, and WSe_2_ areas, with increasing brightnesses. Every triangular unit of TMDs shows an equilateral triangular shape of uniform width, each defined by parallel heterointerfaces, which could be controlled with nanoscale precision. First, the composition (i.e., transition metal and chalcogen constituents) can be independently modulated. SEM images of a WS_2_–MoS_2_–WS_2_ (Figure 1b) and WSe_2_–WS_2_–WSe_2_ (Figure 1c) demonstrate the transition metal and chalcogen modulation, respectively. Photoluminescence (PL) and Raman spectra further confirm the compositional modulation (Figure S1). Second, the lateral dimension (i.e., width) of these LDHs can be controlled with a precision of a few nanometers. Figure 1d shows WS_2_–WSe_2_–WS_2_ LDHs with WSe_2_ widths (labeled as *d*) of 180, 45, 15, and 5 nm. These crystalline LDHs were synthesized using a modulated metal–organic chemical vapor deposition (MOCVD) process, which enables independent control over growth duration and precursor delivery to achieve precise composition and dimension control (see Supporting Information Note S1 and Figure S2).

**Figure 1:**
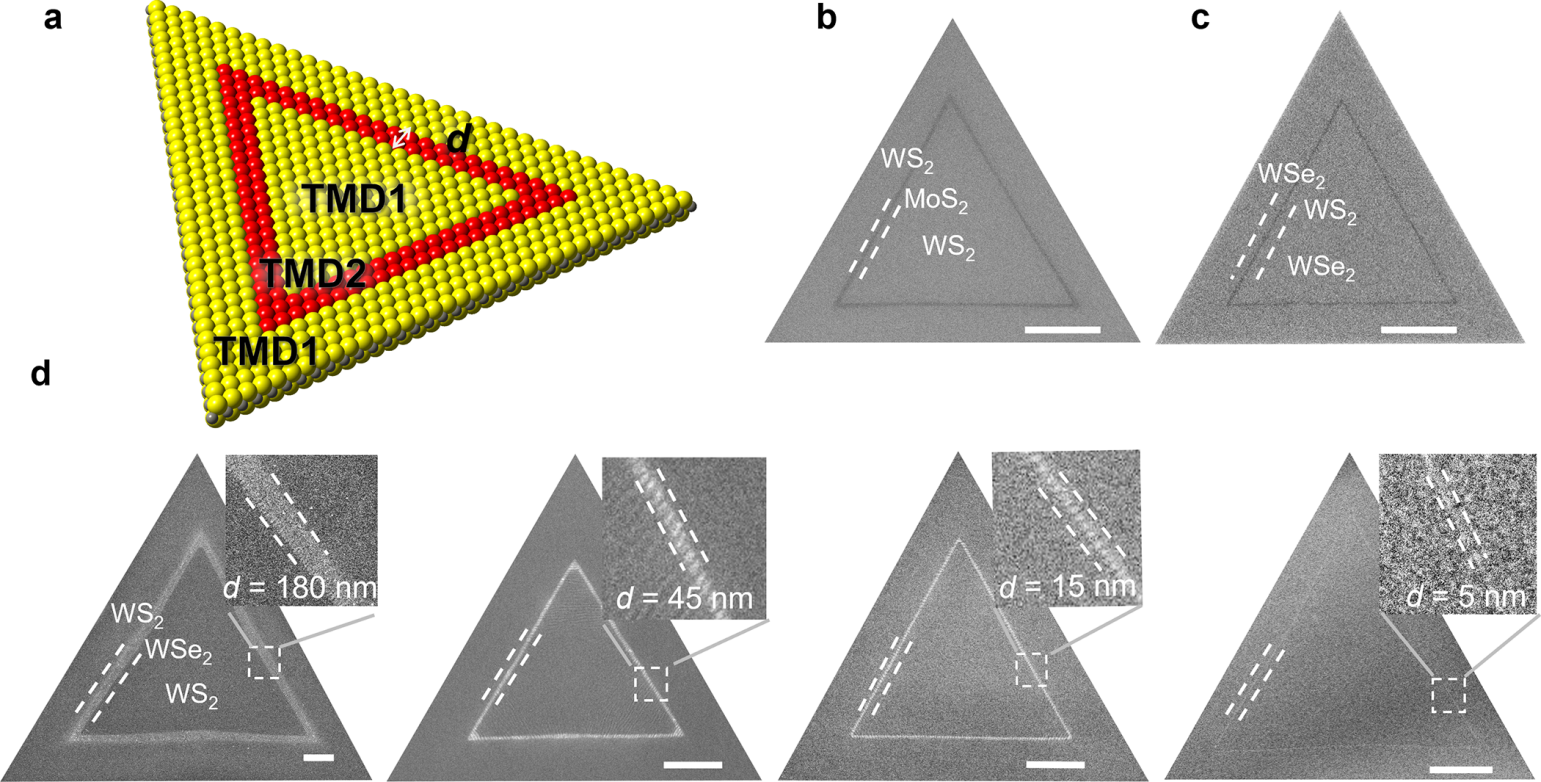
**TMD lateral double heterostructures with controlled composition and dimension. a,** Schematic of TMD lateral double heterostructures, where the width of the middle TMD is *d*. **b**, **c**, SEM images of WS_2_–MoS_2_–WS_2_ and WSe_2_–WS_2_–WSe_2_ LDHs. **d**, SEM images of WS_2_–WSe_2_–WS_2_ LDHs with width of WSe_2_ *d* = 180, 45, 15, and 5 nm, respectively. The insets show enlarged areas near WSe_2_. Scale bars 1 μm.

The composition modulation and lattice coherence of our TMD LDHs were confirmed by energy-dispersive X-ray spectroscopy (EDX) and high-angle annular dark-field scanning transmission electron microscopy (HAADF-STEM). The HAADF-STEM image of a representative WS_2_–WSe_2_–WS_2_ (with *d* = 20 nm, transferred onto a lacey carbon grid) shows brighter triangular lines corresponding to the WSe_2_ area (Figure 2a). EDX elemental mappings of the same LDH reveal that tungsten is uniformly distributed, while selenium (sulfur) contrast increases (decreases) within the WSe_2_ region (Figure 2b), confirming the composition modulation in this WS_2_–WSe_2_–WS_2_ LDH.

**Figure 2:**
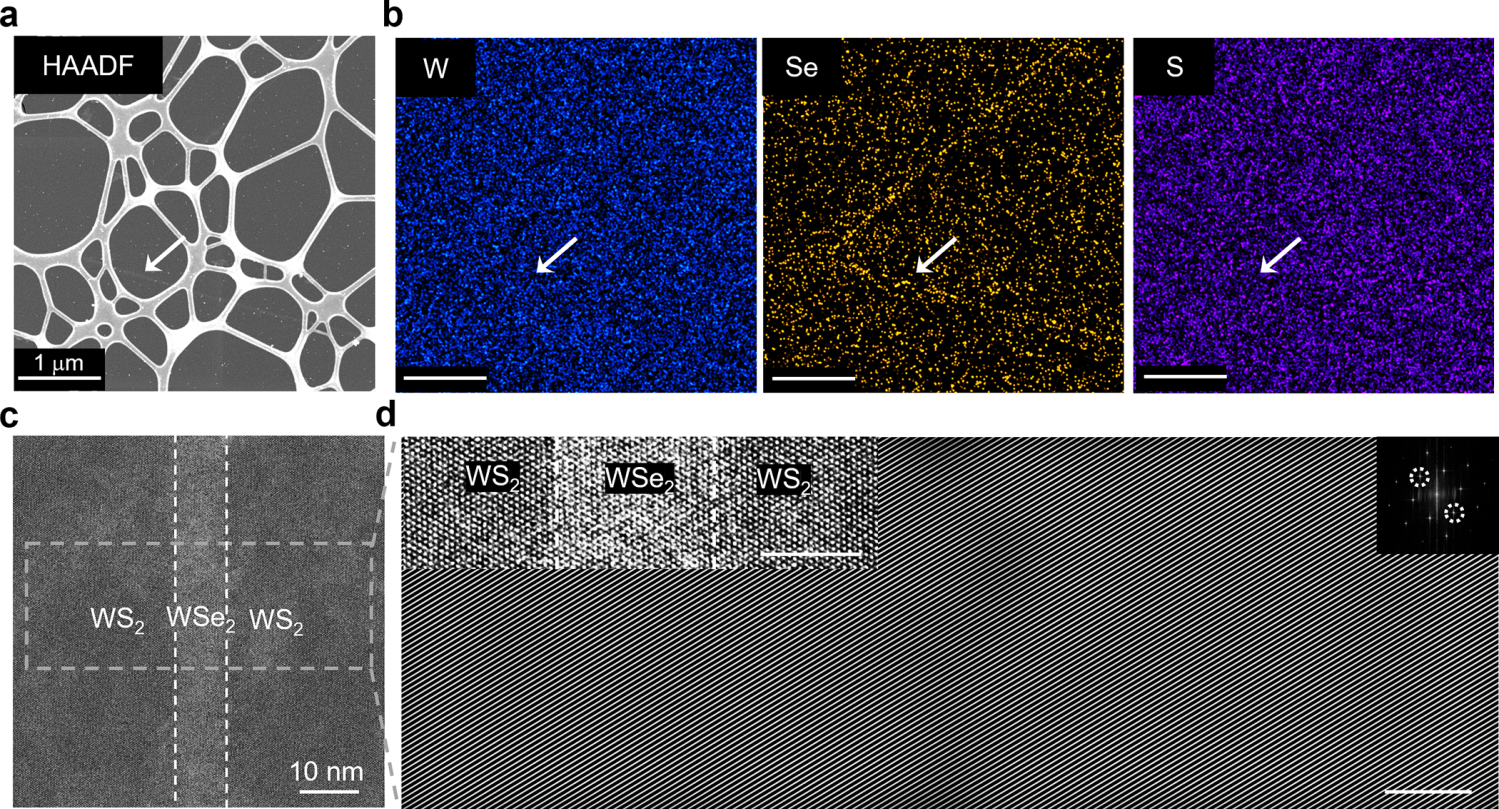
**Coherent interface of WS**_**2**_ **-WSe**_**2**_ **-WS**_**2**_ **lateral double heterostructures**. **a**, HAADF-STEM image of a WS_2_–WSe_2_–WS_2_ LDH with *d* = 20 nm. **b,** Energy-dispersive X-ray spectroscopy (EDX) elemental mappings of W, Se, and S based in the same field of view in (**a**), where the W map shows uniform contrast while Se and S maps show clear intensity modulations across the WSe_2_ area as highlighted by the white arrows. **c**, HAADF-STEM image near the heterointerface of a WS_2_–WSe_2_–WS_2_ LDH with *d* = 8 nm. The two interfaces in this LDH are indicated by the two white dash lines. **d,** An enlarged junction area (upper left inset) and inverse FFT of the HAADF-STEM image across the LDH for the dashed-box region in (**c**), based on the circled spots in its FFT (upper right inset). Scale bar 5 nm.

These WS_2_–WSe_2_–WS_2_ LDHs maintain lattice coherence without forming misfit dislocations at the heterointerfaces, despite a large lattice mismatch of ∼ 4% between WS_2_ and WSe_2_.^39^ HAADF-STEM images of a WS_2_–WSe_2_–WS_2_ LDH with *d* = 8 nm (Figure 2c) reveal sharp, straight interfaces between WS_2_ (darker contrast) and WSe_2_ (brighter contrast) regions. The HAADF-STEM data taken from a zoomed-in area (Figure 2d upper left) with atomic resolution show continuous lines of atoms with no misfit dislocations near the heterointerface across ∼ 125 unit cells [Figure 2d, demonstrated after the inverse fast Fourier transform (FFT)]. For incoherent heterointerfaces with a 4% lattice mismatch, one dislocation is expected every ∼ 25 unit cells; therefore, these HAADF-STEM data confirm the lattice coherence of our TMD LDHs.^39^ Furthermore, WS_2_–WSe_2_–WS_2_ LDHs show uniform out-of-plane ripples in the WSe_2_ region, consistent with the compressive strain in WSe_2_ due to lattice coherence (Figure S3).

TMD LDHs with nanometer-scale dimensional control offer an ideal platform for investigating dimension-dependent carrier transport mechanisms in monolayer TMD heterostructures. Using WS_2_–WSe_2_–WS_2_ LDHs with varying WSe_2_ width (*d*) as a model system, we systematically studied the electrical transport mechanism of LDHs with different *d*. We fabricated 20 LDH devices with varying *d* ranging from 8 to 180 nm, while maintaining a fixed channel length (2 μm) and width (3 μm) (Figure 3a). The transfer curve of a representative WS_2_–WSe_2_–WS_2_ LDH device with *d* = 8 nm (Figures 3b, S4) shows increasing conductance with larger positive gate voltage (*V*_GS_), resembling a *n*-type field-effect transistor (FET) behavior. Its output curve (Figure 3c) shows a linear current dependence with drain-source voltage (*V*_DS_), indicating an Ohmic contact between the metal electrodes and TMD.

**Figure 3:**
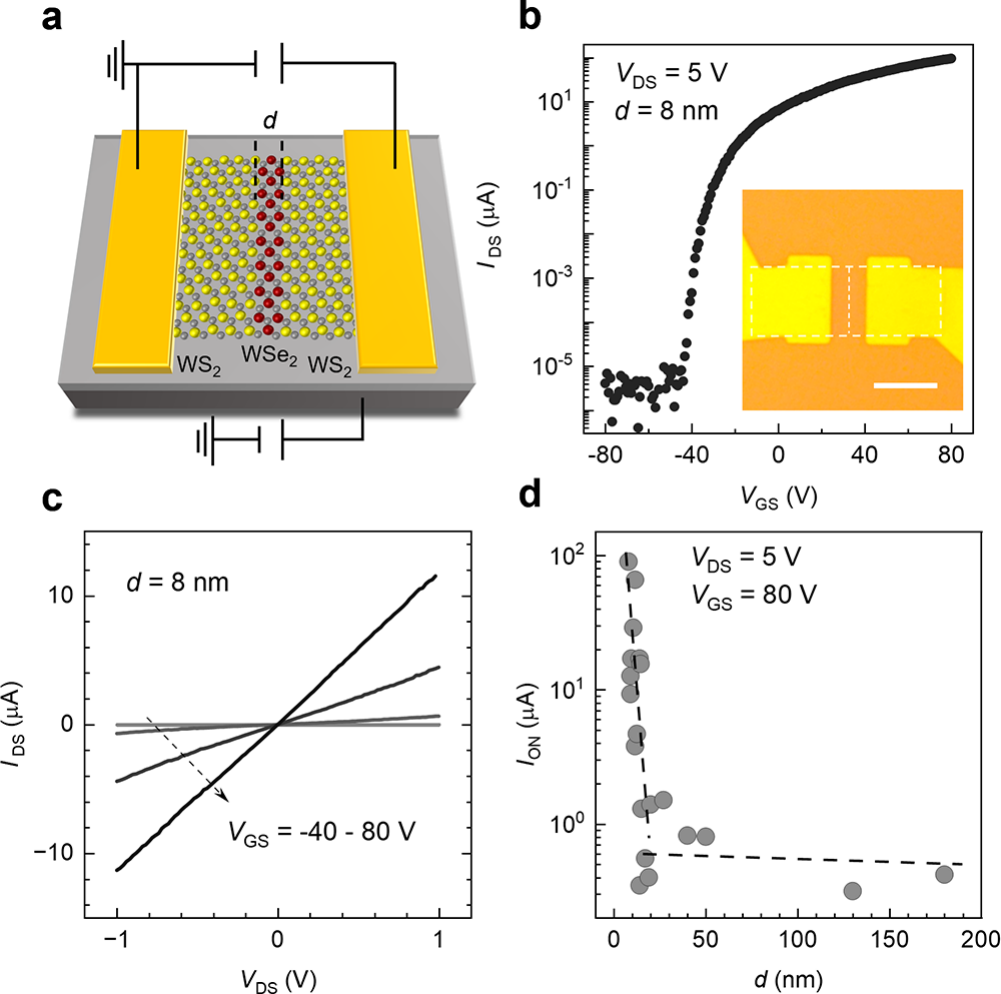
**Dimension-dependent electrical characteristics of WS**_**2**_ **-WSe**_**2**_ **-WS**_**2**_ **devices. a**, Schematic of a two-terminal WS_2_–WSe_2_–WS_2_ LDH device, with WSe_2_ width *d*. **b**, Transfer curve (*I*_DS_-*V*_GS_) of a device with *d* = 8 nm. The inset shows an optical micrograph of a representative device. Scale bar 3 μm. **c**, Output (*I*_DS_-*V*_DS_) curve of the same device in (**b**). **d,** ON*-*state current for LDH devices with different *d*, measured at *V*_DS_ = 5 V, *V*_GS_ = 80 V. Total channel length and width are fixed at 2 and 3 μm, respectively. The ON-state current decreases by nearly 3 orders of magnitude as *d* increases from 8 to 25 nm, while it stabilizes with minimal change as *d* further increases from 25 to 180 nm.

Figure 3d plots the dependence of ON-state (*V*_GS_ = 80 V, *V*_DS_ = 5 V) current of 20 devices on *d* (see Figure S5 for their transfer curves). When *d* < 25 nm, the ON-state current decreases by nearly 3 orders of magnitude as *d* increases from 8 to 25 nm. For *d* > 25 nm, the ON-state current plateaus with minimal variation as *d* further increases from 25 to 180 nm. The total resistance of an LDH device can be expressed as

$$R_{total}=R_{c}+R_{WS2}+R_{WSe2}+R_{S-Se}+R_{Se-S}$$ 1

where *R*_c_, *R*_WS2_, and *R*_WSe2_ are the resistances of metal-WS_2_ contacts, WS_2_ area, and WSe_2_ area, respectively, and *R*_S–Se_ and *R*_Se–S_ are the resistance of WS_2_–WSe_2_ heterojunction and WSe_2_–WS_2_ heterojunction. As *d* increases, *R*_c_ should stay unchanged, and *R*_WS2_ + *R*_WSe2_ should increase linearly with *d*. Therefore, the nearly 3 orders of magnitude decrease of ON-state current when *d* increases from 8 to 25 nm is attributed to resistance changes of the heterojunctions (i.e., *R*_S–Se_ + *R*_Se–S_). While the electron transport across a WS_2_–WSe_2_ interface is expected to experience an energy barrier due to the type-II band alignment,^40–42^ the distinct ON-state current dependence on *d* for *d* < 25 nm and *d* > 25 nm indicates different carrier transport mechanisms in these two regimes, consistent with a barrier whose height and width vary with *d*.

To uncover the different carrier transport mechanisms of LDHs in the large and small *d* regimes, we carried out temperature-dependent electrical transport measurements on two devices, denoted as [Large-*d*] and [Small-*d*], representing the large (∼200 nm) and sub-10 nm (∼8 nm) *d* regimes, respectively. A four-terminal device configuration is employed to eliminate the impact of the metal contacts (Figure 4a). The current–voltage (*I–V*) characteristics of [Large-*d*] between 100 and 160 K exhibit strong temperature dependence (Figure 4b), indicating thermionic transport behavior. This behavior is well-described by the 2D Richardson-Schottky (R-S) model.^43,44^ It is confirmed by the linear dependence of ln *(I)* on $\sqrt{V}$ (Figure 4c), as expected by the R-S equation:

$$I=A_{0}A^{*}T^{1.5}\exp\left[\frac{-\left(\Phi_{B0}-\sqrt{q^{3}V/4\pi \varepsilon_{0}\varepsilon_{r}s}\right)}{k_{b}T}\right]$$ 2

where *A*_0_ is the area of junction, A* is the 2D effective Richardson constant, *ε*_r_ is the permittivity of the barrier (WSe_2_), *ε*_0_ is the vacuum permittivity, *q* is the electron charge, and *s* is the width of the barrier. *T, k*_b_, Φ_B0_, and *s* are the temperature, Boltzmann constant, barrier height, and barrier width of the WS_2_–WSe_2_ interface (see Supplementary Note S2 for more details). These data allow for determination of the zero-bias barrier height (Φ_B0_) for electron transport at the WS_2_–WSe_2_ heterointerface. To calculate Φ_B0_, we first determined *I*_0_ as a function of temperature by linearly extrapolating the ln (*I*) – $\sqrt{V}$ dependence to *V* = 0 V.^45^ Based on extrapolated *I*_0_ between 90 and 170 K, Figure 4d shows the Arrhenius plot of ln­(*I*_0_ */T* ^1.5^) versus 1000/*T* which follows a linear dependence denoted by the dashed line. From the slope of ln­(*I*_0_ */T* ^1.5^) *versus 1/T*, Φ_B0_ in [Large-*d*] was estimated to be 113 meV (Figure 4d). This estimated Φ_B0_ is consistent with the order of magnitude of the previously reported value.^40^ We note that our calculated barrier height, Φ_B0_, is expected to be underestimated, for the following reasons. First, Φ_B0_ was calculated based on transport data at temperatures between 90 and 170 K, as the device transport at *T* > 170 K is dominated by the negative temperature-dependent mobility of WS_2_ which accounts for 80% of the total channel length (Figure S6). This can lead to underestimated Φ_B0_ as thermionic field emission may also contribute to the overall electron transport.^44^ Second, these transport data were taken at *V*_GS_ = 40 V, which is larger than *V*_GS_ = 0 V at which Kelvin probe force microscopy measurements (KPFM) were performed (Figure S3). The larger *V*_GS_ in the transport measurements can lead to an underestimated value of barrier height determined by KPFM (this *V*_GS_ dependence of barrier height is further discussed in Figure 5c).

**Figure 4:**
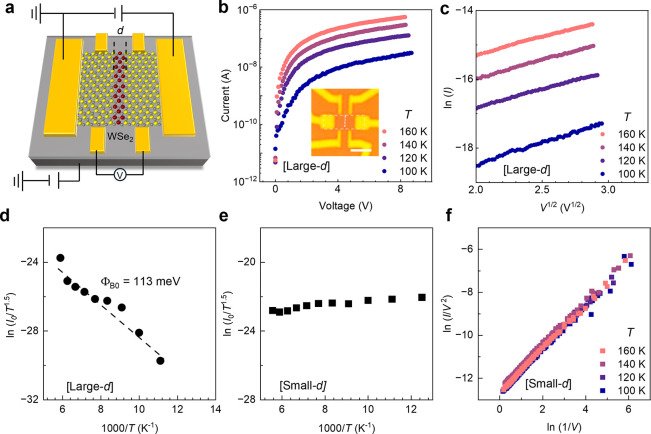
**Low-temperature transport characteristics of two devices [Large-** *d* **] and [Small-** *d* **]. a,** Schematic of a four-terminal WS_2_–WSe_2_–WS_2_ LDH device. **b**, Current–voltage (*I–V)* curves of [Large-*d*] at various temperatures. The inset shows an optical micrograph of a representative device, where a red dash line indicates the location of WSe_2_. Scale bar 2 μm. **c**, Plot of ln *I* - *V* ^1/2^ at various temperatures. **d**, Arrhenius plot of ln (*I*_0_ */T* ^1.5^) – 1000/*T* of [Large-d], where the slope of the linear fitting gives Φ_B0_/*k*_b_ *T*. *I*_0_ is extrapolated at *V* = 0 from (**c**) following the thermionic Richardson-Schottky equation. **e**, Arrhenius plot of ln (*I*_0_ */T* ^1.5^) – 1000/*T* of [Small-d], showing no temperature dependence. The linearly fitted slope represents Φ_B0_/*K*_b_ *T*. **f**, Plot of ln (*I/V* ^2^) – ln (*1/V*) of [Small-d] at various temperatures, where the linear dependence confirms direct-tunneling as the transport mechanism. All data in this figure were measured under gate voltage *V*_GS_ = 40 V. The values of vertical axes in (**c - f**) were calculated based on the following units: *I* and *I*_0_ (A), *T* (K), *V* (V).

**Figure 5:**
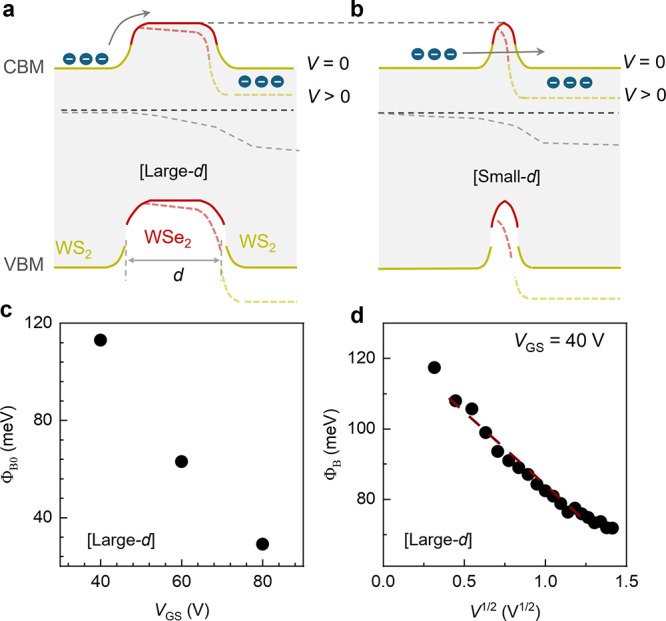
**Electron transport mechanisms in WS**_**2**_ **-WSe**_**2**_ **-WS**_**2**_ **LDHs with [Large-** *d* **] and [Small-** *d* **]. a, b** Energy band diagram of WS_2_–WSe_2_–WS_2_ LDHs with [Large-*d*] (**a**) and [Small-*d*] (**b**) *d*. At [Large-*d*], electron transport across the energy barrier is dominated by thermionic processes. At [Small-*d*], electron transport across the energy barrier is dominated by direct tunneling processes, which is facilitated by narrow WSe_2_ and the formation of depletion region. **c,** Dependence of barrier height on *V*_GS_ of [Large-d]. **d**, Plot of barrier height - *V* ^1/2^ of [Large-d], showing dependence in agreement with image charge effect.

In contrast, [Small-*d*] shows different transport characteristics. The Arrhenius plot (Figure 4e) shows a temperature-independent ln­(*I*_0_/*T* ^1.5^) despite the presence of an energy barrier for electron (Figure S7), indicating that the electron transport is dominated by tunneling. The linear dependence of ln­(*I*/*V* ^2^) on ln­(1/*V*) (Figure 4f) confirms that the electron transport mechanism in [Small-*d*] is direct tunneling rather than Fowler–Nordheim tunneling^46–48^ (see Supplementary Note S2 for more details).

To further probe the transition, we performed temperature-dependent transport measurements on a LDH with intermediate WSe_2_ barrier width (*d* ∼ 40 nm). The *I–V* characteristics (Figure S8a) exhibit a weaker temperature dependence compared to the [Large-*d*] device. Thermionic fitting yields a smaller effective barrier height of 36 meV at *V*_GS_ = 40 V (Figure S8b), compared to 113 meV for [Large-*d*]. This reduction in Φ_*B0*_ suggests that tunneling contributes appreciably to the total current together with thermionic transport, consistent with the expectation that both transport mechanisms coexist at intermediate barrier widths.

The above temperature-dependent electrical transport data confirm that [Large-*d*] follows thermionic transport while [Small-*d*] follows direct tunneling transport. Such different transport mechanisms are schematically shown by their band diagrams. In [Large-*d*] devices, the tunneling current is (exponentially) small due to the wide energy barrier for electrons; therefore, thermionic current dominates the transport (Figure 5a). In [Small-*d*] devices, the narrow barrier width, combined with band modulation by the formation of the depletion region, promotes electron-tunneling-dominated transport (Figure 5b). We note that the observed direct tunneling in [Small-*d*] is analogous to the tunneling behavior in conventional Esaki diodes, in which the depletion region (serving as the tunnel barrier) is typically around 10 nm.^49^ The tunneling behavior in [Small-*d*] is a combination of a narrow WSe_2_ width (small *d*) and the formation of depletion regions in WS_2_ and WSe_2_. Such depletion regions are expected to be wider compared to those in metal–semiconductor-metal (MSM) FET structures due to lower carrier densities in semiconductor LDHs. As a result, the effective barrier width in our LDHs is narrower than that of the MSM devices, leading to an enhanced electron tunneling across the barrier.

Moreover, the barrier height of electron transport Φ_B0_ can be tuned electrostatically. As *V*_GS_ increases from 40 to 80 V, the calculated Φ_B0_ decreases from 113 to 28 meV (Figure 5c). This barrier reduction at larger *V*_GS_ is consistent with their band alignment transitioning from *npn* toward *n* ^*+*^ *nn* ^*+*^. As *V*_GS_ increases, the electron concentration in WS_2_ and WSe_2_ increases, resulting in upward shifts of their Fermi levels. Due to the n- and p- type doping for WS_2_ and WSe_2_, respectively, the Fermi level shifts more in WSe_2_, resulting in a smaller Φ_B0_. Additionally, the barrier height (Φ_B_) at nonzero bias is tunable based on applied bias. Figure 5d shows the linear dependence of Φ_B_ on $\sqrt{V}$ , consistent with the thermionic transport described by the R-S equation incorporating the image charge effect.^50^ These results highlight the unique tunability of transport in 2D double heterostructure.

In summary, we have demonstrated the synthesis of monolayer TMD LDHs with controlled composition and sub-10 nm dimensional control, including WS_2_–MoS_2_–WS_2_ and WS_2_–WSe_2_–WS_2_. These LDHs exhibit coherent interfaces without misfit dislocations, despite their large lattice mismatch. Electrical transport measurements reveal a transition from thermionic emission (for [Large-*d*]) to direct tunneling (for [Small-*d*]) transport mechanism as the WSe_2_ width decreases. Furthermore, the energy barrier for thermionic transport can be tuned by the gate voltage and drain bias. These results demonstrate the unique modulated-MOCVD synthesis capabilities of TMD heterostructures and advanced engineering of carrier transport in 2D systems. These findings provide new insights for future development of transistors (e.g., tunneling FET with high tunneling current density) and complex heterostructures based on monolayer 2D heterostructures.

## Supplementary Material



## Data Availability

All data needed to evaluate the conclusions are presented in the paper and/or the Supporting Information. Additional data related to this paper are available from the corresponding author upon reasonable request.

## Notes

Synthesis and characters; extraction of electron energy barriers; PL and Raman characteristics; AFM and KPFM characteristics; electrical characteristics; band alignment of pure TMDs and heterostructures (PDF)
