# Supplementary material for: Nanoscale Control of Carrier Transport in Monolayer Transition-Metal Dichalcogenide Double Heterostructures
Source: Nano Lett. 2026 May 8;26(19):6349–55. doi: 10.1021/acs.nanolett.6c00504 (PMC13195730; doi:10.1021/acs.nanolett.6c00504)
Supplement: Supplementary file 1 [file nl6c00504_si_001.pdf]

Supporting Information

Nanoscale Control of Carrier Transport in Monolayer Transition Metal Dichalcogenide Double Heterostructures

Jinpeng Tian[1], Guangming Cheng[2], Jingtao Tan[3], Satya Butler[3], Yin Liang[1], Haining Mao[1], Jaehoon Ji[1], Jaerin Kim[1], Nan Yao[2], Saien Xie[1,2]*

[1]Department of Electrical and Computer Engineering, Princeton University, Princeton, New Jersey 08544, USA.

[2]Princeton Materials Institute, Princeton University, Princeton, New Jersey 08544, USA.

[3]Department of Mechanical and Aerospace Engineering, Princeton, New Jersey 08544, USA.

*Corresponding author

Note S1. Synthesis and characterization: *TMD LDHs growth:* Synthesis of monolayer TMD LDHs was conducted in a home-built MOCVD system with a 2-inch quartz tube furnace (Figure S1a). Tungsten hexacarbonyl (THC), molybdenum hexacarbonyl (MHC), diethyl sulfide (DES), and dimethyl selenide (DMSe) were selected as chemical precursors for W, Mo, S and Se, respectively. All precursors were kept in bubblers at a constant pressure of ~850 Torr and introduced into the furnace with Ar as the carrier gas at room temperature. All precursors were introduced into the growth furnace through individual mass flow controllers (MFCs) for precise regulation of their flow rates. Growth of the TMD LDHs occurred at a constant temperature of 680 ^oC and a total pressure of ~1.8 Torr throughout. Flow rates of all precursors were kept constant during its growth, 4 sccm of THC or MHC with Ar as the carrier gas, 1 sccm DES or DMSe with Ar as the carrier gas, 5 sccm H_2 and 350 sccm Ar. The LTDHs grew the corresponding precursors step-by-step (Figure S1b, c). To achieve sub-10 nm precision, we optimized the precursor concentration to maintain a low growth rate (~24 nm/min). This low growth rate allows the WSe_2 width to be precisely manipulated by adjusting the growth duration in increments of seconds. The interface sharpness is enabled by a 1-minute purging step between chalcogen precursor switching. During this purging step, the S or Se flow is terminated while the W precursor and carrier gas flow are maintained. The growth time of different LDHs and width is summarized in (Table S1).

Device fabrication: All devices were fabricated with as grown heterostructures on 285 nm SiO_2/P^{++} Si substrates. We first pattered e-beam lithography marks and located the position of the TMD LDHs by SEM. Then 2-terminal and 4-terminal device electrodes were defined using standard e-beam lithography in a Raith EBPG 5150 Plus with PMMA 950 A4 as the e-beam photoresist, followed by e-beam evaporation of 10 nm Bi/30 nm Au. After lift-off using acetone, we defined the device channel and etched unwanted TMD regions with O_2 plasma etching in an Oxford PlasmaPro 80 RIE system.

Transport measurements: Electrical transport measurements were conducted in Janis ST-500 cryogenic vacuum probe station under ~10^{-6} torr pressure with liquid N_2 coolant. 2-terminal and 4-terminal measurements were performed with a Keysight B1500A semiconductor device parameter analyzer.

SEM and STEM characterization: SEM measurements were conducted in an FEI Verios 460 system with Immersion mode, 5 kV accelerating voltage, and 25 pA beam current.

Atomic resolution high-angle annular dark-field (HAADF) STEM imaging and energy dispersive X-ray spectroscopy (EDX) mappings were performed on a Titan Cubed Themis 300 double Cs corrected scanning/transmission electron microscope (S/TEM), equipped with an extreme field emission gun source and a super-X EDS system. The system was operated at 300 kV.

Raman and Photoluminescence (PL) spectroscopy: Raman spectroscopy of monolayer TMD LDHs flakes is conducted on a confocal Raman system (LabRAM ARAMIS-Raman Spectrometer, Horiba Scientific) with a 532 nm laser. The emitted PL and Stokes Raman signals were collected by 600 and 1800 lines per mm grating, respectively.

Note S2. Extraction of electron energy barriers: The TMD lateral double heterostructures (TMD LDHs) can be regarded as two PN diodes connected back-to-back. At higher temperatures, thermally activated charge carriers can overcome the barrier height (Φ_B) of TMD1-TMD2, resulting in thermionic emission. If the barrier height is low enough, then the thermionic emission can occur at lower temperatures. The *I-V* characteristics in this regime can be modeled using the simplified 2D R-S equation[1] for thermionic emission

$$I = A_0 A^* T^{1.5} exp\left[\frac{-\left(\Phi_{B0} - \sqrt{\frac{q^3 V}{4\pi\varepsilon_0\varepsilon_r s}}\right)}{k_b T}\right] \quad (1)$$

where A_0 is the area of the junction, $A^* = q(8\pi k_B^3 m^*)^{0.5}/h^2$ is the Richardson constant for a 2D system (m^*, electron effective mass), T is the temperature, k_B is Boltzmann constant, q is the elementary charge, and Φ_{B0} is the effective electron barrier height at a given gate–source voltage V_{GS}.

When the temperature decreases, there may not be enough thermal energy for the charge carriers to overcome the barrier height, and the charge injection is dominated by tunneling through the interface barrier. With increasing bias voltage, the shape of the tunnel barriers changes from a direct tunneling mechanism to Fowler-Nordheim (F-N) tunneling[2–4]:

At low bias, direct tunneling

$$I \propto V exp[-\frac{2s\sqrt{2m_*\Phi_{B0}}}{\hbar}] \quad (2)$$

At high bias, Fowler Nordheim (F-N) tunneling

$$I \propto V^2 exp[-\frac{4s\sqrt{2m_*\Phi_{B0}}^3}{3\hbar qV}] \quad (3)$$

Where $\hbar$ is Planck's constant divided by 2π and m_* is the effective mass of the charge carrier, s is the barrier width.

The above *I-V* relations in eq. (2) and eq. (3) can be linearized on a logarithm scale to become eq. (4) and eq. (5), respectively:

Direct tunneling

$$\ln(\frac{I}{V^2}) \propto \ln(\frac{1}{V}) - \frac{2s\sqrt{2m_*\Phi_{B0}}}{\hbar} \quad (4)$$

Fowler Nordheim (F-N) tunneling

$$\ln(\frac{I}{V^2}) \propto -(\frac{1}{V})\frac{4s\sqrt{2m_*\Phi_{B0}}^3}{3\hbar q}] \quad (5)$$

Therefore, we can separate the direct tunneling and F-N tunneling through plotting $\ln(I/V^2)$ vs. $\ln(1/V)$ and $\ln(I/V^2)$ vs. $1/V$. In the case of F-N tunneling, $\ln(I/V^2)$ vs. $1/V$ will show a negative linear dependence, and in the case of direct tunneling, $\ln(I/V^2)$ vs. $\ln(1/V)$ will show positive linear dependence. In our [Small-*d*] device, reduced barrier thickness due to depletion region promotes direct tunneling. This is emphasized by Figure S9, which shows no obvious negative slope (as would be expected from eq. (5) for F-N tunneling).

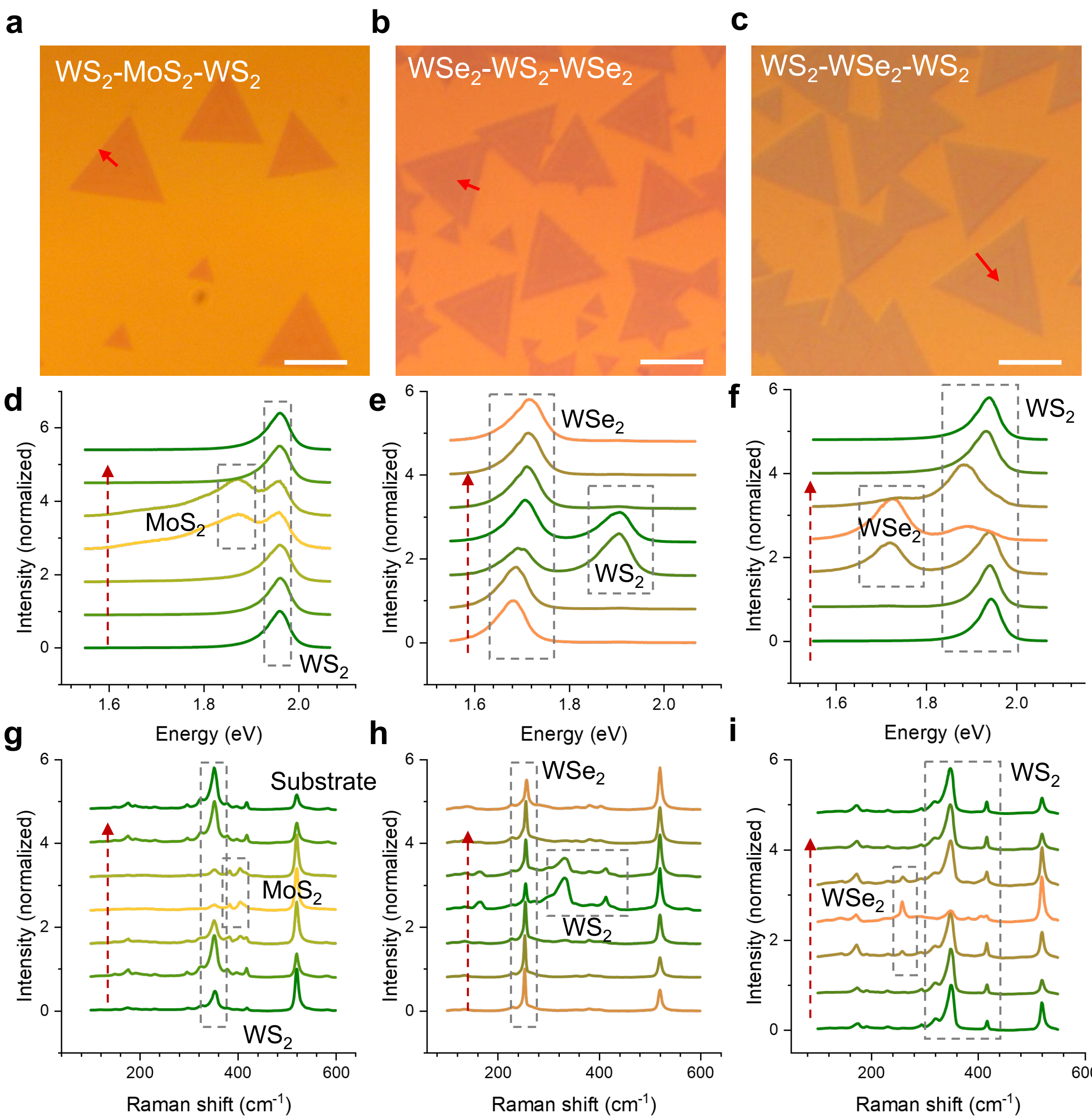

Figure S1. PL and Raman characteristics of TMD LDHs. a, b, c Optical image of WS_2-MoS_2-WS_2, WSe_2-WS_2-WSe_2 and WS_2-WSe_2-WS_2, respectively. Scale bar 10 µm. **d, e, f** and **g, h, i** show line scan PL and Raman shifts along lines shows in (**a**), (**b**), and (**c**), respectively.

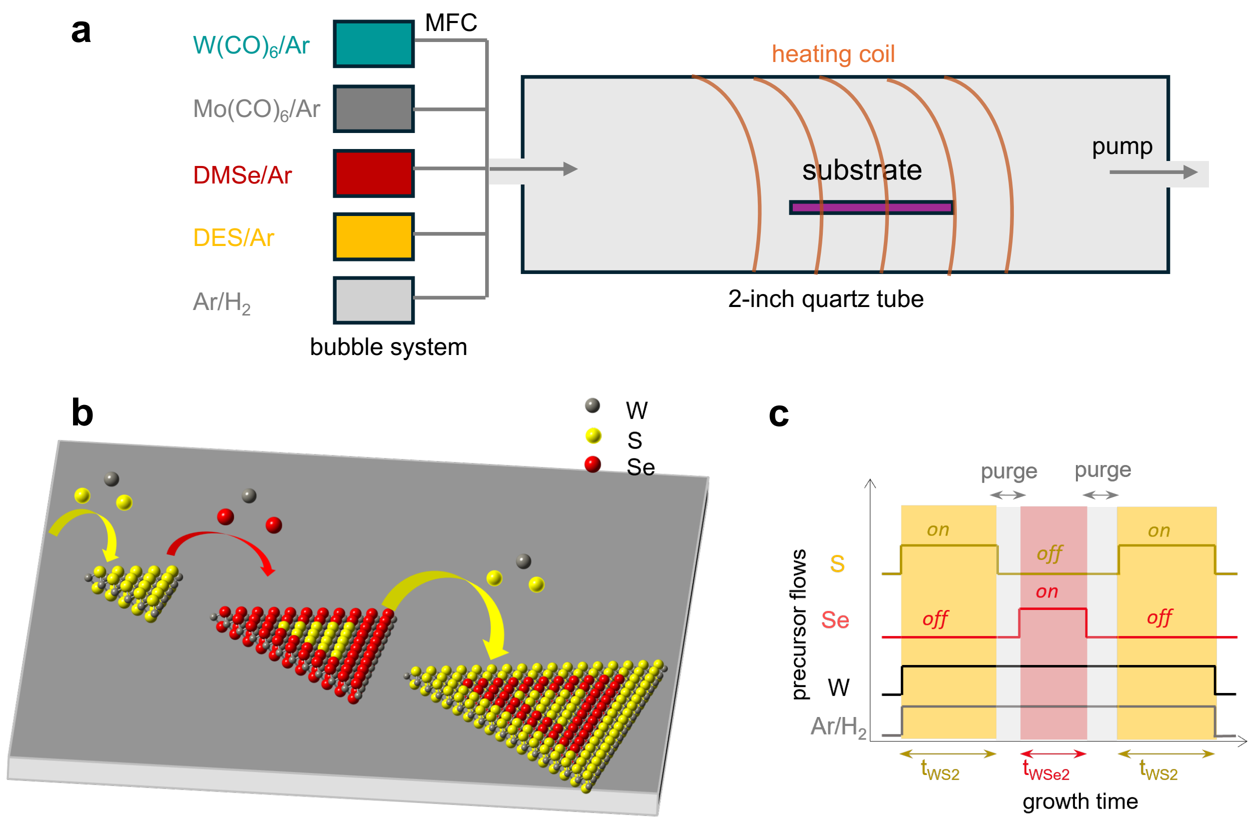

Figure S2. Schematic of MOCVD system and growth sequence of TMD LDHs. a, Schematic of a 2-inch home-built MOCVD system. All precursors are installed in bubblers with Ar as carrier gas. **b**, Schematic of the growth sequence for LTDHs. First step for WS_2 core, second step for WSe_2, third step for WS_2. **c**, Precursor flows throughout the TMD LDH growth process in (**b**).

Table S1 | Summary of the growth time of each step for TMD LDHs in **Figure 1**.

LDH	TMD1	Purge	TMD2	Purge	TMD1
WS_2-MoS_2-WS_2	1 h	5 min	30 s	5 min	1 h
WSe_2-WS_2-WSe_2	1 h	1 min	30 s	1 min	1 h
WS_2-WSe_2-WS_2 $d = 180$ nm	1 h	1 min	5 min	1 min	1 h
WS_2-WSe_2-WS_2 $d = 45$ nm	1 h	1 min	2 min	1 min	1 h
WS_2-WSe_2-WS_2 $d = 15$ nm	1 h	1 min	50 s	1 min	1 h
WS_2-WSe_2-WS_2 $d = 5$ nm	1 h	1 min	20 s	1 min	1 h

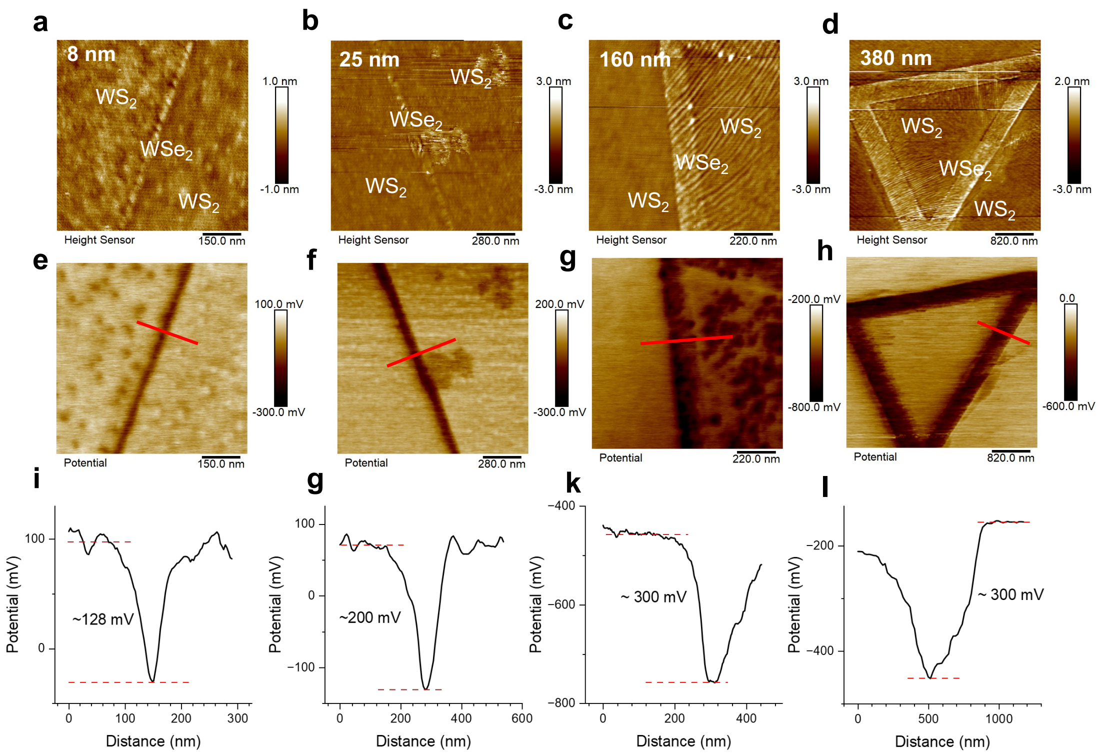

Figure S3. AFM and KPFM characteristics of WS_2-WSe_2-WS_2 LDH with different *d*. a-d, AFM height images of d = 8, 25, 160, 380 nm WS_2-WSe_2-WS_2 interfaces, respectively. **e-h** and **i-l** show the corresponding KPFM images and potential profiles (along the red line of corresponding KPFM images), respectively. The differences of surface potential between WS_2 and WSe_2 are 128, 200, 300, and 300 meV for d = 8, 25, 160, and 380 nm LDHs, respectively.

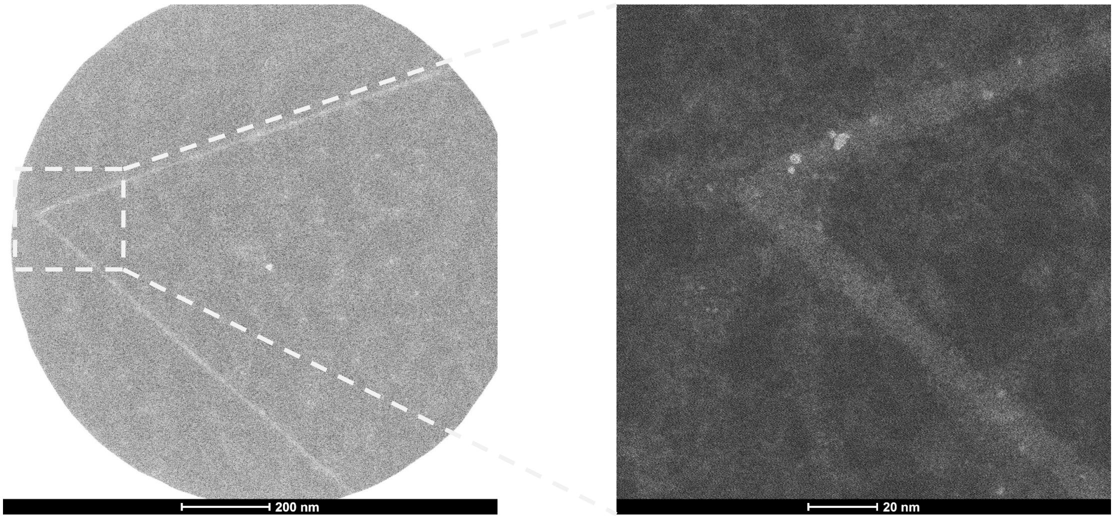

Figure S4. HAADF-STEM image of a WS_2-WSe_2-WS_2 LDH with d = 8 nm. A HAADF-STEM image near the heterointerfaces and an enlarged area outlined by the dashed box.

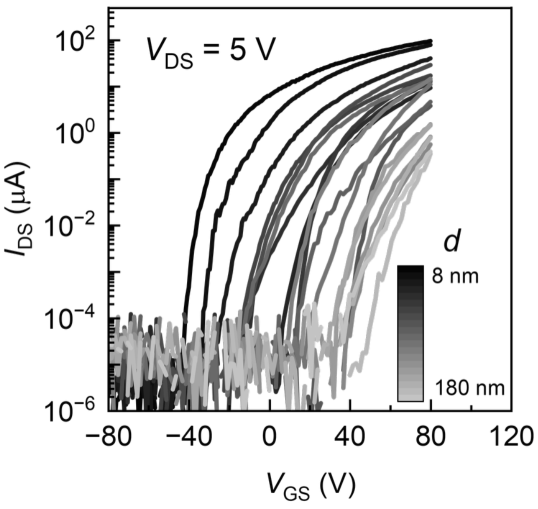

Figure S5. Transfer curve of 2-terimial device in Figure 3d with different *d*.

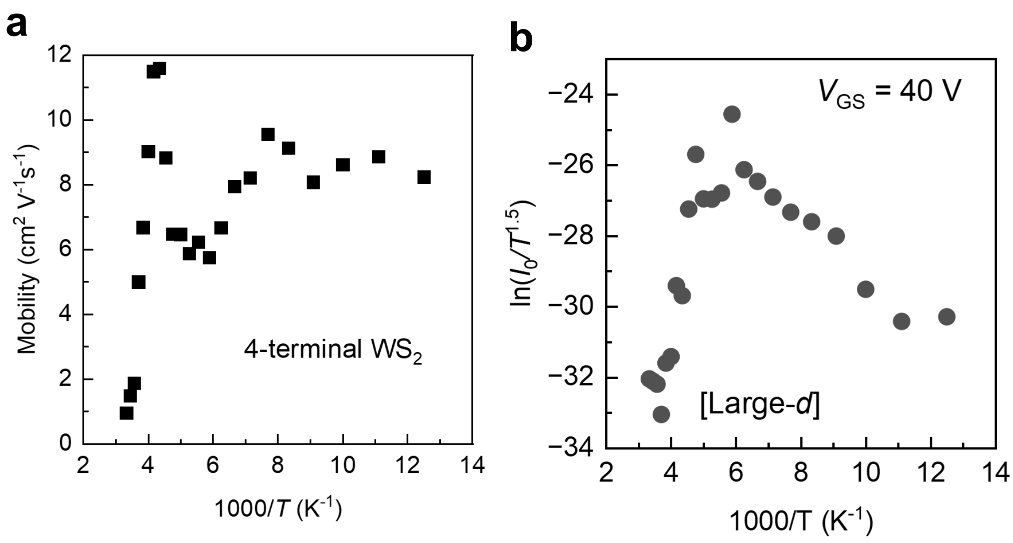

Figure S6. Electrical characteristics of WS_2 and LDH. a, WS_2 mobility vs. 1000/*T*, showing that the WS_2 varies significantly above 170 K. **b,** Arrhenius plot of $\ln(I_0/T^{1.5})$ versus 1000/*T* of [Large-*d*] device. The measured LDH device currents at >170 K are dominated by the WS_2 mobility's negative temperature dependence, which accounts for 80% of the total channel length. This can lead to underestimated Φ_{B0} as thermionic field emission may also contribute to the overall electron transport.

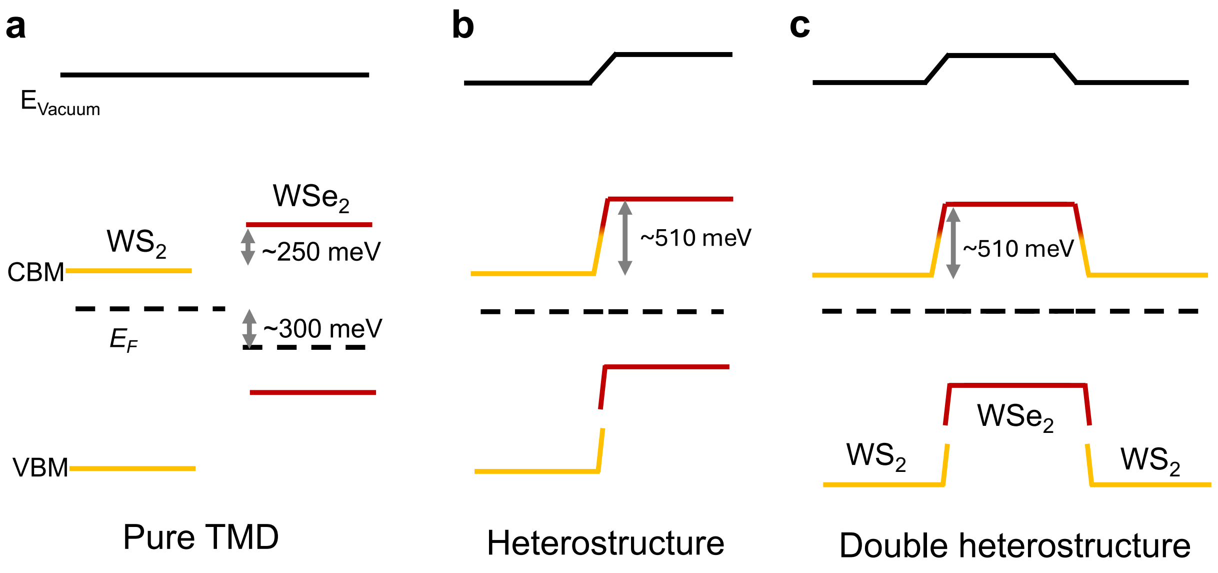

Figure S7. Band alignment of pure TMDs and heterostructures. **a**, band alignment of pure WS_2 and WSe_2. The electron affinity difference between pure WS_2 and WSe_2 is ~ 250 meV[5]. And the difference of work function between WS_2 and WSe_2 is ~300 meV (see **Figure S3 for KPFM data**). **b**, Band alignment of WS_2 and WSe_2 in single junction heterostructure. **c**, Band alignment of WS_2-WSe_2-WS_2 double heterostructures.

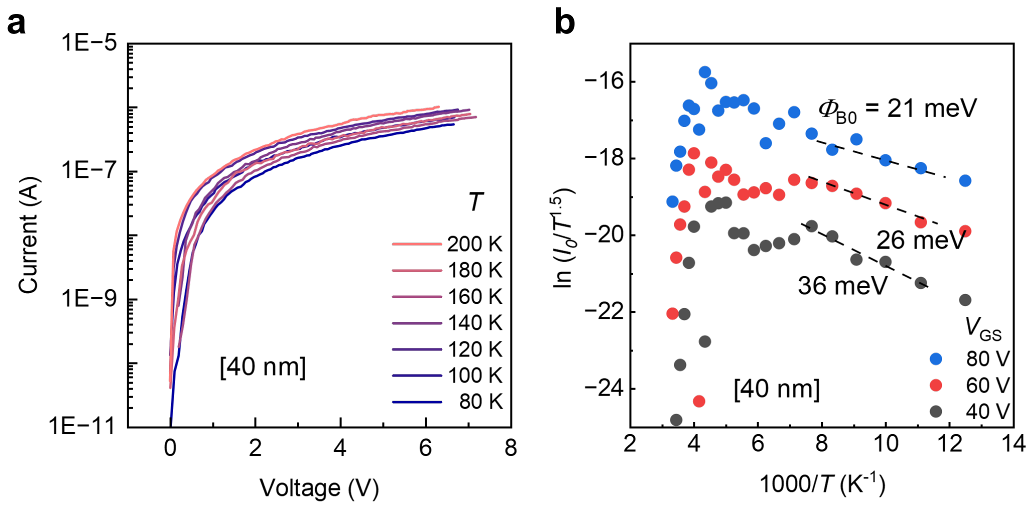

Figure S8. Low-temperature transport characteristics of *d* ~ 40nm device. a, Current-voltage (*I-V*) curves of [*d* ~ 40 nm] at various temperatures. The I-V shows weak temperature dependence. **c**, Arrhenius plot of ln ($I_0/T^{1.5}$) - 1000/T of [*d* ~ 40nm] under various V_{GS}. The values of vertical axes in (c) were calculated based on the following units: I and I_0 (A), T (K), V (V).

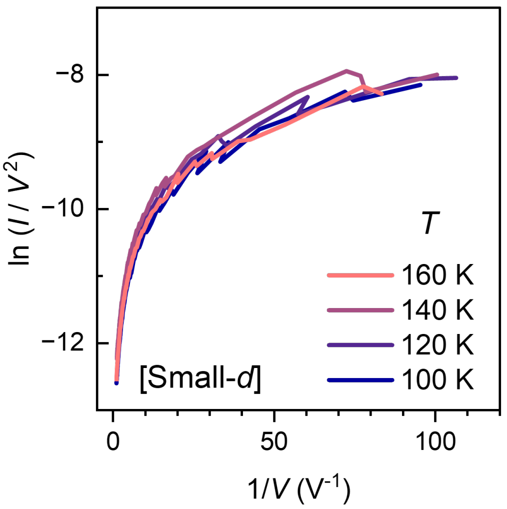

Figure S9. ln(I/V^2) vs. 1/V plot of [Small-d] according to the F-N tunneling linearized equation. The positive slope suggests the tunneling is not F-N tunneling dominated.

References

(1) Shen, P.-C.; Su, C.; Lin, Y.; Chou, A.-S.; Cheng, C.-C.; Park, J.-H.; Chiu, M.-H.; Lu, A.-Y.; Tang, H.-L.; Tavakoli, M. M.; Pitner, G.; Ji, X.; Cai, Z.; Mao, N.; Wang, J.; Tung, V.; Li, J.; Bokor, J.; Zettl, A.; Wu, C.-I.; Palacios, T.; Li, L.-J.; Kong, J. Ultralow Contact Resistance between Semimetal and Monolayer Semiconductors. *Nature* **2021**, *593* (7858), 211–217. https://doi.org/10.1038/s41586-021-03472-9.

(2) Araidai, M.; Tsukada, M. Theoretical Calculations of Electron Transport in Molecular Junctions: Inflection Behavior in Fowler-Nordheim Plot and Its Origin. *Phys. Rev. B* **2010**, *81* (23), 235114. https://doi.org/10.1103/PhysRevB.81.235114.

(3) Sarker, B. K.; Khondaker, S. I. Thermionic Emission and Tunneling at Carbon Nanotube–Organic Semiconductor Interface. *ACS Nano* **2012**, *6* (6), 4993–4999. https://doi.org/10.1021/nn300544v.

(4) Wang, W.; Lee, T.; Reed, M. A. Mechanism of Electron Conduction in Self-Assembled Alkanethiol Monolayer Devices. *Phys. Rev. B* **2003**, *68* (3), 035416. https://doi.org/10.1103/PhysRevB.68.035416.

(5) Herbig, C.; Zhang, C.; Mujid, F.; Xie, S.; Pedramrazi, Z.; Park, J.; Crommie, M. F. Local Electronic Properties of Coherent Single-Layer WS2/WSe2 Lateral Heterostructures. *Nano Lett.* **2021**, *21* (6), 2363–2369. https://doi.org/10.1021/acs.nanolett.0c04204.
